# Characterization of the oral and gut microbiome in children with obesity aged 3 to 5 years

**DOI:** 10.3389/fcimb.2023.1102650

**Published:** 2023-03-29

**Authors:** Ting Ma, Zeyu Wu, Jing Lin, Chao Shan, Aisaiti Abasijiang, Jin Zhao

**Affiliations:** ^1^ Department of Cariology and Endodontics, The First Affiliated Hospital of Xinjiang Medical University, The Affiliated Stomatology Hospital of Xinjiang Medical University, Urumqi, China; ^2^ Stomatology Disease Institute of Xinjiang Uyghur Autonomous Region, Xinjiang Medical University, Urumqi, China

**Keywords:** oral microbiome, gut microbiome, obesity, high-throughput sequencing, preschool children

## Abstract

The ever-increasing global prevalence of obesity has trended towards a younger age. The ecological characteristics and changes of the oral and gut microbial community during childhood are poorly understood.In this study, we analyzed the salivary and fecal microbiota of 30 children with obesity and 30 normal weight children aged 3-5 years *via* third-generation long-range DNA sequencing,with the aim of understanding the structure of childhood microbiota and identifying specific oral and gut microbial lineages and genera in children that may be associated with obesity.The results revealed significant variation in alpha diversity indices among the four groups (Chao1: *P* < 0.001; observed species: *P* < 0.001; Shannon < 0.001). Principal coordinate analysis (PCoA) and nonmetric multidimensional scaling (NMDS) revealed significant differences in oral and gut microbial community structure between obesity and controls. The Firmicutes/Bacteroidetes (F/B) abundance ratios of oral and intestinal flora among children with obesity were higher than those of controls. The most abundant phyla and genera found in oral and intestinal flora were Firmicutes, Proteobacteria, Bacteroidetes, *Neisseria*, *Bacteroides*, *Faecalibacterium*, *Streptococcus*, *Prevotella* and so on. Linear discriminant analysis effect size (LEfSe) revealed higher proportions of *Filifactor* (LDA= 3.98; *P* < 0.05) and *Butyrivibrio* (LDA = 2.54; *P* < 0.001) in the oral microbiota of children with obesity, while the fecal microbiota of children with obesity were more enriched with *Faecalibacterium* (LDA = 5.02; *P* < 0.001), *Tyzzerella* (LDA=3.25; *P* < 0.01), *Klebsiella* (LDA = 4.31; *P* < 0.05),which could be considered as dominant bacterial biomarkers for obesity groups.A total of 148 functional bacterial pathways were found to significantly differ in the oral and gut microbiota among controls and obesity using PICRUSt 2. Most predicted functional pathways were clustered in biosynthesis. In conclusion, This work suggests there were significant differences in oral and gut microbiota in controls and obesity groups, microbiota dysbiosis in childhood might have significant effect on the development of obesity.

## Introduction

Despite growing recognition of obesity as a major public health crisis, obesity rates continue to increase worldwide. In 2016, 1.9 billion people were overweight worldwide (Benahmed et al., 2021). Data released by the World Health Organization (WHO; http://www.who.int/mediacentre/factsheets/fs311/en/) reported that more than 340 million children and adolescents aged 5–19 in addition to 41 million children under the age of 5 were overweight or obese (NCD Risk Factor Collaboration, 2017). The rising prevalence of obesity has caused a significant increased in the incidence of other obesity-related comorbidities, such as type 2 diabetes (Lingvay et al., 2022), hypertension (El Meouchy et al., 2022), non-alcoholic fatty liver disease (Hagström et al., 2021), cardiovascular disease (Hariharan et al., 2022), and coronavirus 2019. (COVID-19) as described by Huang et al., (Huang et al., 2020). Being obese during childhood and adolescence was not only found to adversely affect health in later life (Singh et al., 2008) but also negatively impact psychosocial wellbeing (Quek et al., 2017) and led to lower educational attainment (Caird et al., 2014). As such, identifying the causes of childhood obesity is important in the context of preventative health. Although genetics and lifestyle directly influence the onset and progression of obesity (Mohammadian et al., 2022), recent studies have confirmed that oral and gut microbiomes play a crucial role in the pathogenesis of obesity (Araujo et al., 2020; Pinart et al., 2021).

The gut is considered the “base camp” of microbes within the human body (Mizutani et al., 2020). The total number of microorganisms in this habitat exceeds 10^14^, or 10 times the number of human somatic cells (Sender et al., 2016). The oral cavity is the second largest site of bacterial colonization in the human body after the gut (Willis and Gabaldón, 2020). The oral and gut microbiomes play important roles in human health, and an imbalance between these populations often leads to oral and systemic disease.

The oral cavity is the point of entry for many pathogens, and this cavity is closely related to the human microbiota (Irrazábal et al., 2014; Said et al., 2014; Garrett, 2015). Changes in the oral microbiome reportedly result in conditions such as dental caries, periodontitis, and systemic illness (Zhang et al., 2022). For example, a higher salivary abundance of certain bacterial species, such as *Streptococcus mutans* and *Scardovia wiggsiae*, was identified in samples obtained from caries-affected adolescents (Eriksson et al., 2018). The presence of *Porphyromonas gingivalis*, *Prevotella intermedia*, and *Actinomycetes* in the subgingival biofilm and saliva is considered a biomarker of periodontitis (Maciel et al., 2016; Belstrøm, 2020). The presence of *Fusobacterium* and *Pseudomonas gingivalis* in the oral cavity is also reportedly associated with colorectal and pancreatic cancer (Zheng et al., 2021). Importantly, the oral microbiome is reportedly associated with the pathogenesis of obesity as well (Abu-Shawish et al., 2022). Saliva samples obtained from overweight women exhibited a higher proportion of *Selenomonas noxia* (>1.05% of total oral bacteria), which was identified as a reliable biomarker for weight-related conditions (Goodson et al., 2009).

Constituents of the gut microbiota also have been implicated in the pathogenesis of obesity (Henao-Mejia et al., 2012). While lean mice harboring gut microbes transplanted from obese mice exhibited weight gain, monozygotic mice harboring a “leaner” microbiome did not (Turnbaugh et al., 2006; Ridaura et al., 2013). Similarly, fecal bacterial transfer from obese to germ-free mice increased fat deposition and metabolic complications in the germ-free group, suggesting an association between gut microbiome composition and the pathogenesis of obesity (Backhed et al., 2004; Lee et al., 2020). Injection of *Porphyromonas gingivalis* into mice resulted in a reduction in the number gut Bacteroidetes as well as increased systemic inflammation and insulin resistance, demonstrating that the oral flora can modulate the gut microbiome (Arimatsu et al., 2014). Thus, the oral–gut axis plays a significant role in the development of digestive system diseases or conditions related to digestion and metabolism.

The abovementioned studies confirmed that the composition of oral and gut bacterial communities directly or indirectly contributes to the development of obesity. However, little is known regarding the effects of these microbial populations on obesity in young children. The microbiota continues to develop through childhood, and gaining excess weight during childhood is likely to lead to lifelong overweight and obesity. Thus, childhood may be the critical time for microbiota interventions to promote good health or prevent disease (Chen et al., 2020). As such, it is crucial to understand the structures and functions of the pediatric oral and gut microbiota. To date, few studies have examined the microbiome of preschool children aged 3 to 5 years, and current findings regarding differences in the oral and gut microbial composition in obese children are inconsistent. As the prevalence of obesity has trended toward children of younger ages, it is essential to elucidate the relationship between obesity and the pediatric microbiota.

This study utilized third-generation PacBio long-read sequencing to analyze the oral and gut microbial composition based on samples obtained from 30 children with obesity and 30 healthy children. Specific bacteria associated with childhood obesity were identified, and the relevant functional microbial pathways were elucidated. These results broaden our current understanding of obesity-associated microecological dysbiosis and provide a foundation for predicting, preventing, and treating childhood obesity.

## Materials and methods

### Ethics statement

This study was approved by ethical committee of the First Affiliated Hospital of Xinjiang Medical University (20170214-162). We obtained written informed consent from all participants (legal parent or guardian).

### Participants

A total of 60 children were recruited from 16 kindergartens in eight districts of Urumqi, China. Thirty children with obesity were chosen for the experimental group and 30 normal weight children were considered controls. In this study, a sample size similar to those previously reported was evaluated (Mervish et al., 2019; Chen et al., 2020; Balakrishnan et al., 2021). Inclusion criteria included several parameters: (1) children aged 3-5 years; (2) children not using orthodontic devices; (3) children without gastrointestinal complaints, such as constipation or diarrhea; and/or (4) childhood obesity as diagnosed based on the Centers for Disease Control (CDC) guidelines (https://www.cdc.gov/obesity/data/childhood.html ) with subject BMI ≥ 95^th^ percentile of average (subjects with a BMI between the 5^th^ and 85^th^ percentiles were classified as controls). Children with gingivitis and periodontitis were excluded. Children treated with antibiotics within the three months prior to sample collection or who would not cooperate were also excluded.

### Clinical parameters

With the help of a parent or guardian, each child completed a questionnaire to provide socio-demographic and nutritional data. Sugar consumption habits (SHC) were determined based on reported frequencies of consumption of sugary foods, sugary drinks and sweetened milk/yogurt/tea/coffee (Quan et al., 2018). SHC was recorded as either low (total score of 3 to 8), medium (total score of 9 to 14), or high (total score of 15 to 18). Scoring criteria followed a specific scale: (1) = seldom/never; (2) = 1-3 times a month; (3) = once a week; (4) = 2-6 times a week; (5) = once a day; and (6) = more than once a day. Cariological examination was performed on each subject at kindergarten under natural light by a qualified dentist to assess for the presence of caries in addition to decayed, missing, and filled teeth (dmft); debris index (DI) was also determined (Kappa coefficient ≥ 0.8). Physical examination entailed assessment of participant height and weight to calculate body mass index (BMI;kg/m^2^). Participants were classified as either controls or obesity according to BMI as previously described.

### Samples collection

Saliva samples were collected according to the protocol described by the Human Microbiome Project (https://www.hmpdacc.org/hmp/, Mcinnes and Cutting, 2010). Saliva samples were obtained prior to the group breakfast in the kindergarten (no water or food intake for at least 8h prior to the breakfast). At least 10mL of non-irritating whole saliva was collected in a sterile centrifuge tube that was stored at -80°C within 2h of sample collection. Fecal samples were obtained from children using a Fecal Microbial Genome Protection Kit. Samples were divided into three aliquots and stored at -80°C until further analysis. Groups were classified according to individual BMI and sample type: (1) NW-o (oral microbiota of normal weight children), (2) NE-g (gut microbiota of normal weight children), (3) Ob-o (oral microbiota of children with obesity); and (4) Ob-g (gut microbiota of children with obesity).

### DNA extraction and PCR amplification

Genomic DNA was extracted using the Omega Mag-bind soil DNA kit (M5635-02) according to manufacturer instructions. DNA concentrations and integrity were evaluated using a NanoDrop spectrophotometer and 2% agarose gel electrophoresis. Amplification of bacterial 16S rRNA was performed using PCR with an NEB Q5 High-Fidelity polymerase (M0491L). Selected primers were F: AGAGTTTGATCMTGGCTCAG; R: ACCTTGTTACGACTT. The target fragment obtained *via* PCR amplification were cut and recycled using an AXYGEN gel recovery kit. The amplicon mixture was proportionally pooled according to fluorescence quantitative results and sequencing requirements for each sample.

### Library preparation and DNA sequencing

Sequencing libraries were prepared using reagent in PacBio’s Template Prep Kit 1.0. DNA fragments were paired-end sequenced using the PacBio platform. Raw sequencing data were saved in FASTQ format. Microbiome bioinformatics were performed as previously described using QIIME 2 2019.4 (Bolyen et al., 2019) with slight modifications implemented according to official tutorials (https://docs.qiime2.org/2019.4/tutorials/). Briefly, raw sequence data were demultiplexed using the demux plugin followed by primer cutting using the cutadapt plugin (Martin, 2011). Sequences were then quality filtered, denoised, merged and chimera removed using the QIIMEDADA2 denoise-paired plugin (Callahan et al., 2016). Sequence quality control was performed using DADA2 with resultant amplicon sequence variant (ASV) clustering at 100% similarity. Singleton ASVs were removed and non-singleton ASVs aligned using MAFFT, and a phylogeny was constructed using FastTree 2. Taxonomy was assigned to ASVs using the classify-sklearn naiüve Bayes taxonomy classifier in the feature-classifier plugin against 16S-NTASV reference sequences. The above procedure was performed by Shanghai Personalbio Company. Sequencing data have been uploaded to the NCBI (National Center for Biotechnology Information) sequence read archive (BioProject no. PRJNA903817).

### Bioinformatic analysis

**Figure 1:**
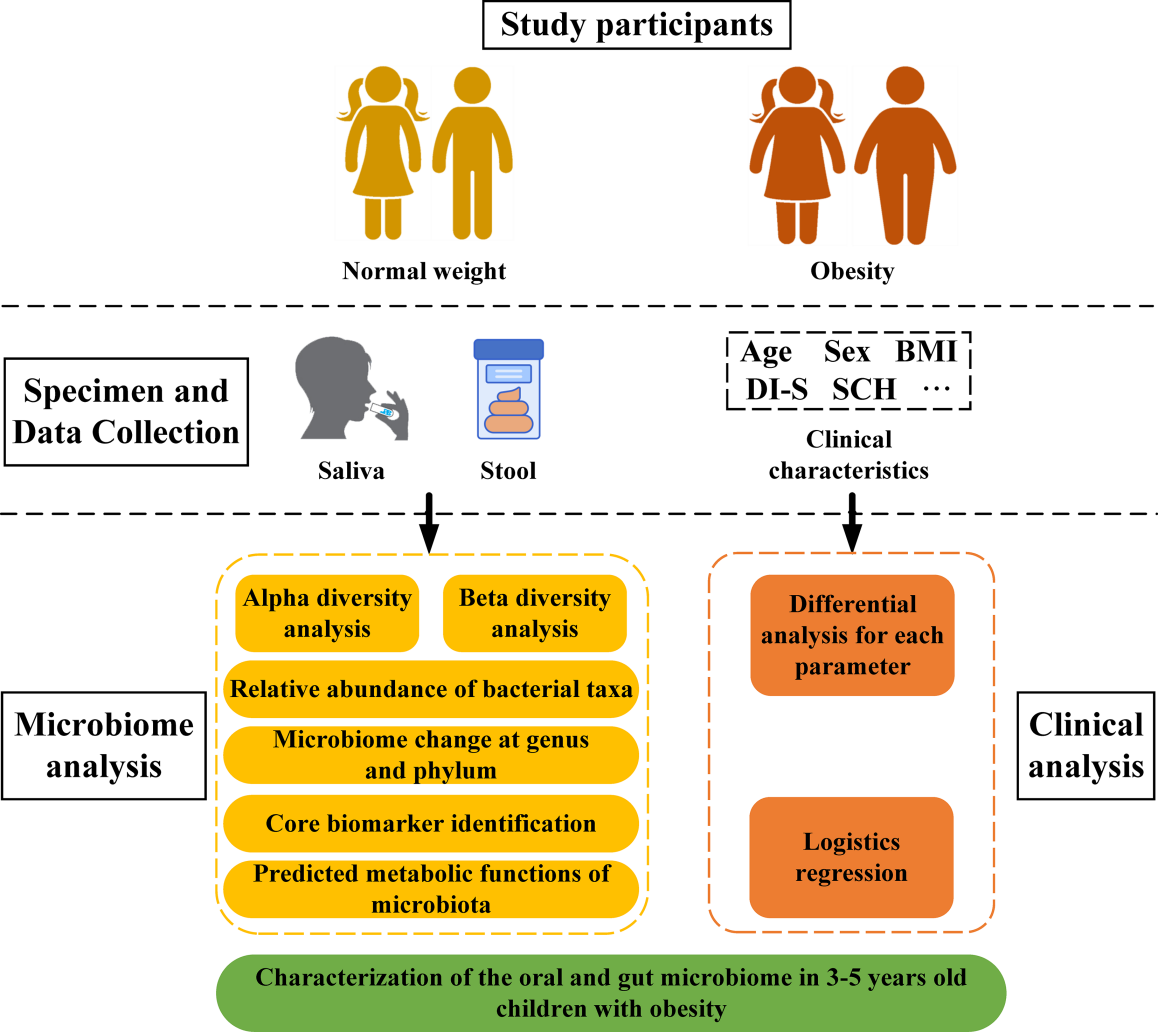
Study flowchart.

After removal of singleton ASVs, sample composition on all six classification levels (phylum, class, order, family, genus, and species) were visualized using QIIME2 (2019.4). Alpha diversity metrics (Chao1, observed species, Shannon, Simpson, Faith’s Phylogenetic Diversity, Pielou’s evenness index and Good’s coverage) were calculated using R software and the ggplot2 package. For visualization of beta diversity among groups, unweighted and weighted UniFrac distances were calculated and plotted using principal coordinate analysis (PCoA) and non-metric multidimensional scaling (NMDS). Results were displayed using R software (vegan package). Linear discriminant (LDA) and effect size (LEfSe) analyses were performed to concurrently and differentially analyze all classification levels. The LDA value was set to 2 to facilitate robust, statistically significant identification of different species and marker species between groups. PICRUSt2 was utilized to predict bacterial community functions using MetaCyc as a functional database. Data relevant to the 16S rRNA sequencing pipeline as well as clinical parameter analysis are shown in **Figure 1**.

### Statistical analyses

Mann-Whitney U, Student’s t or chi-squared tests were applied according to data distribution for comparison of continuous and categorical variables. The Wilcoxon signed-rank test was used to test significance and identify the group with the highest abundance of each species in taxa composition analysis. Alpha diversity comparisons were performed using Kruskal-Wallis and Dunn tests. Fisher’s exact test was used to compare parameters. Kruskal–Wallis and Wilcoxon tests were used to perform LEfSe. *P* values < 0.05 after correction for the Benjamini-Hochberg false discovery rate were considered to be significant. All tests were two-sided, and *P* < 0.05 was considered statistically significant.

## Results

### Demographic and clinical parameters

No significant differences in age, sex, oral cavity features, or sugar consumption among normal weight controls and children with obesity were found (*P* > 0.05). The BMI index values were found to be greater in obesity subjects as compared to controls (*P* < 0.05; **Table 1**).

**Table 1:** Demographic and clinical characteristics.

Variable	Univariate analysis			Multivariate analysis ^d^		
	Normal weight Control (n=30)	Obesity (n=30)	*P*	OR	95%CI	*P*
Age (mean, deviation)	4.87, 0.58	4.84, 0.68	0.45 ^a^	0.26	0.02-3.48	0.31
Sex (male, %)	19, 63.30%	18, 60.00%	0.79 ^b^	1.11	0.11-15.17	0.93
BMI (mean, deviation)	15.00, 1.15	20.12, 2.74	0.03 ^a^	5.26	1.82-15.17	0.002
DIS (0, %;1, %; 2, %)	10, 33.3%; 14, 46.7%; 6, 20.0%	5, 16.7%; 16, 53.3%; 7, 23.3%	0.12 ^c^	2.45	0.10-58.49	0.401
Dental caries (caries, %)	9, 30%	11, 36.7%	0.59 ^b^	0.35	0.01-12.35	0.564
dmft (mean, deviation)	3.47, 3.64	3.03, 3.93	0.72 ^a^	0.82	0.48-1.39	0.456
SCH (low, %; medium, %)	27, 90%; 3, 10%	23, 76.7%; 3, 16.7%	0.15 ^c^	48.77	0.56-4254.65	0.234
Height (mean, deviation)	111.79, 4.50	112.24, 8.81	0.015 ^a^	0	0	0
Weight (mean, deviation)	18.81, 2.39	26.19, 5.86	0.001 ^a^	3.73E+07	0	0

BMI, body mass index; DIS, debris index simplified; SCH, sugar consumption habits.

a Student’s t test;

b Chi-square test;

c Mann-Whitney U test;

d Multivariate P-value were adjusted for age, sex (male or female), BMI, DIS (0, 1, 2, 3), dental caries (yes or no), dmft, SCH (low, medium, high), Height (cm), Weight (kg).

### Sequencing data

A total of 810,482 original sequences were obtained from 120 samples of 60 children. After quality filtering and denoising, 512,475 valid sequences were obtained (**Supplementary Table 1**). The average number of sequences per sample was 4,271. Sequence lengths mainly ranged from 1400 to 1600 base pairs (**Supplementary Figure 1**), meeting quality requirements. The rarefaction curve eventually flattened, indicating that a continued increase in sequencing depth did not facilitate detection of a large number of novel ASVs; sequencing results were thus determined to have sufficiently reflected sample diversity (**Supplementary Figure 2**).

### Bacterial richness and diversity analysis

**Figure 2:**
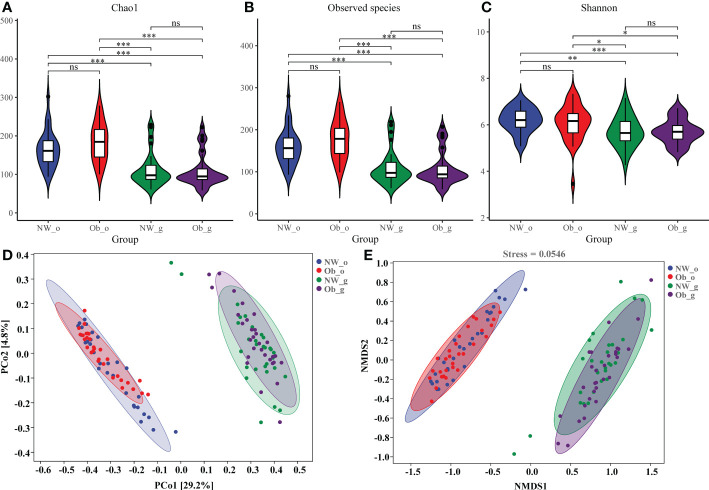
Alpha and beta diversity of microbiota among the four groups. **(A)** A violin diagram of Chao 1; **(B)** Observed-species index data; and **(C)** Shannon index data. The horizontal line within a box represents the median, a dot indicates an observed value, box margins are interquartile ranges (50% of the observations) and whisker lines extend for 1.5 times the interquartile range. Asterisks (*); (**); and (***) represent significant differences at *P* < 0.05, *P* < 0.01, and *P* < 0.001, respectively. **(D)** Principal coordinate analysis (PCoA) data of bacterial communities from the four groups. **(E)** Non-metric dimensional scaling (NMDS) analysis of bacterial β-diversity from the four groups. ns, no significant.

Differences in Chao1, Observed species, and. Shannon and Good’s coverage indices were all statistically significant (*P* < 0.001). Chao1 (**Figure 2A**) and the Observed species 1 index (**Figure 2B**) reflected bacterial community richness, while the Shannon index (**Figure 2C**) comprehensively represented the evenness of the bacterial community richness. Both diversity and richness of the oral microbiome were significantly higher as compared to the gut. While oral microbiota richness in obese children was the highest, the diversity of intestinal microbiota was found to be the lowest. The species coverage of samples evaluated in this study was greater than 99% (**Supplementary Figure 3**), indicating saturation of species diversity at this sequencing depth.

Beta diversity describes the comparison of diversity between different habitats. We used PCoA and NMDS analyses based on weighted-UniFrac values to calculate differences between sample groups. Contribution rates of oral and intestinal flora in children with normal weight and obesity were found to have been 29.2% on PCoA1 (**Figure 2D**). Because the stress value of NMDS analysis was 0.0718, results were confirmed to have been reliable. Differences in composition of oral and intestinal flora were confirmed as shown in **Figure 2E**. However, marked overlap of floral composition among the NW-o and Ob-o groups and the NW-g and Ob-g groups was noted, indicating that children with obesity and controls had similar microbiota compositions in comparable body regions (**Figures 2D,E**). Unweighted-UniFrac PCoA and NMDS data are shown in **Supplementary Figure 4**.

### Oral and gut bacterial community composition in children with obesity and normal weight controls

**Figure 3:**
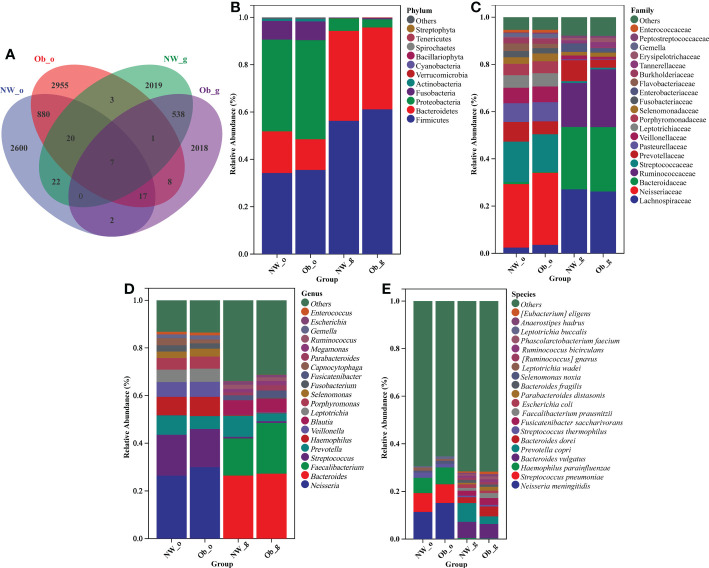
**(A)** Venn diagram. Taxonomic composition and abundance distribution at **(B)** phylum; **(C)** family; **(D)** genus; and **(E)** species levels.

A total of 11,397 ASVs was detected in 120 samples (**Supplementary Table 2**), which were finally divided into 11 phyla, 18 classes, 33 orders, 65 families, 109 genera, and 149 species (**Supplementary Table 3**). **Figure 3A** shows the number of ASVs at different taxonomic levels,most ASVs could be ascribed to genus and species (**Supplementary Figure 5**). As detailed in the Venn diagram (**Figure 3A**), The number of ASVs in different groups were found. Only seven ASVs (less than 0.1%) were common to all groups, suggesting that all groups had less common bacterial species.

According to relative abundance analysis of microbial taxa, differences in the composition of oral and gut microbiota between children with obesity and controls were found. At the phylum level (**Figure 3B**), the dominant bacterial phyla in oral and intestinal samples were Firmicutes, Proteobacteria, and Bacteroidetes, which together accounted for more than 90% of all bacterial flora. A markedly higher abundance of Fusobacteria in the oral cavity as compared to the gut was noted. The oral cavity and gut F/B abundance ratios in children with obesity(2.74 and 1.77, respectively) were higher than those of normal weight children (1.94 and 1.48, respectively).

As shown in **Figure 3C**, family-level taxa dominant in the oral cavity that had a relative abundance of > 5% were Neisseriaceae, Streptococcaceae, Prevotellaceae, Pasteurellaceae, Veillonellaceae, and Leptotrichiaceae. In the gut, Lachnospiraceae, Bacteroidaceae, Ruminococcaceae, and Prevotellaceae accounted for more than 80% of bacteria.

As shown in **Figure 3D**, *Neisseria*, *Streptococcus*, *Prevotella*, *Haemophilus*, and *Veillonella* were the dominant genera found in the oral cavity of both obesity and controls, respectively. *Bacteroides*, *Faecalibacterium*, *Prevotella*, *Blautia*, *Fusicatenibacter*, and *Ruminococcus* accounted for a larger share of genera in gut samples of children with obesity and normal weight controls, respectively.

As shown in **Figure 3E**, the main species in the oral cavity of normal weight and obese children were *Neisseria meningitidis*, *Streptococcus pneumoniae*, and *Haemophilus parainfluenzae*. In addition, samples obtained from the oral cavity of obese group were found to have harbored *Selenomonas noxia* and *Leptotrichia buccalis*.Interestingly,*Selenomonas noxia* was previously reported to potentially serve as a special biomarker for obese women. Several gut bacterial species with a relative abundance > 1% included *Bacteroides vulgatus*, *Prevotella copri*, *Bacteroides dorei*, *Fusicatenibacter saccharivorans*, *Faecalibacterium prausnitzii*, and *Escherichia coli*. What’s more, *Bacteroides fragilis* in the gut of normal weight children in addition to *Parabacteroides distasonis*, *Ruminococcus gnavus*, *Ruminococcus bicirculans*, and *Phascolarctobacterium faecium* in the gut of obese children also exhibited increased relative abundance. No other species were noted to have had a relative abundance > 1%.

**Figure 4:**
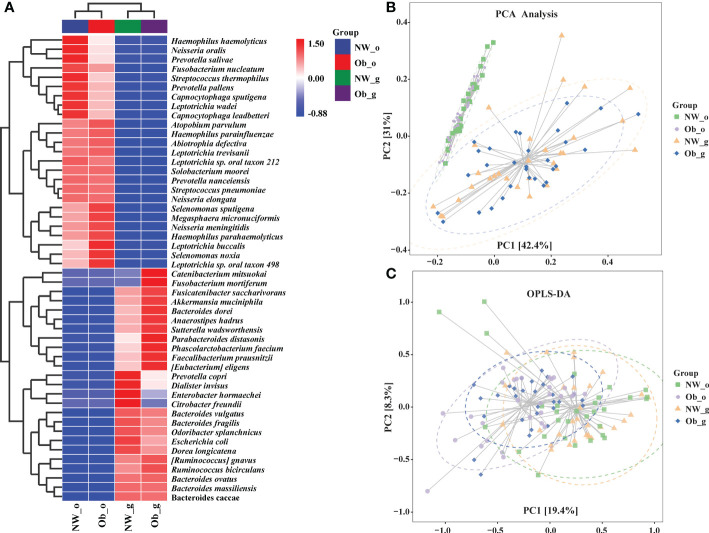
**(A)** Heatmap clustering analysis of bacterial communities among all four groups at the genus level; **(B)** Principle component analysis (PCA); **(C)** Orthogonal Partial Least Squares Discriminant Analysis (OPLS-DA).

As shown in **Supplementary Figure 6**, random forest plot data were generally consistent with those of taxonomic composition analysis. To further describe distribution trends relevant to species abundance, we constructed a heatmap to display species composition. The genus level was the default, weighted pair group method with arithmetic mean (UPGMA) clustering was then performed using the Pearson correlation coefficient matrix of constituent data with findings arranged according to results of clustering analysis. Red represents genera high in abundance while blue represents genera low in abundance, indicating that the presence of highly abundant species among these groups markedly altered microbiome structure in different parts of the human body (**Figure 4A**). Further analyses, including PCA in **Figure 4B** and orthogonal projections to latent structures discriminate analysis (OPLS-DA) shown in **Figure 4C** also revealed differences in the composition of species abundance between samples in the ordination space.

### Core biomarker identification in oral and gut samples of children with obesity and normal weight controls

**Figure 5:**
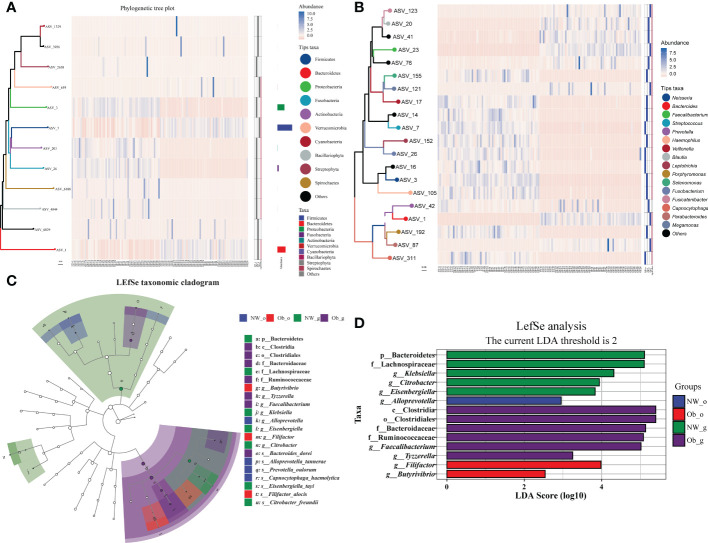
Taxonomic heatmap at the phylum **(A)** and genus **(B)** levels. From left to right: 1) phylogenetic tree map, colored amplicon shared variance (ASV) feature sequences and their connected branches according to taxonomic level; 2) abundance heat map; 3) differential heat map that shows each group with its most abundant species (filled in blue), the significance of the difference between this group and other samples tested; significant differences are denoted with pink as determined *via* the Wilcoxon rank-sum test (considering a false-discovery rate [FDR]-corrected *P* value < 0.05 as significant); 4) FDR correction is “BH” multiple test correction (Benjamini Y. and Hochberg Y, 1995). **(C)** Linear discriminate analysis effect size (LEfSe) taxonomic cladogram. The colored nodes from the inner to outer circles represent the hierarchical relationship of all taxa from phylum to genus levels. Taxa enriched in different groups are shown with different colors; taxa with non-significant changes are colored white. The diameter of each small circle represents taxa abundance. **(D)** Enriched taxa with linear discriminate analysis (LDA) scores >2 are shown in the histogram. The greater the LDA score was, the more significant the phylotype microbiota was in comparison.

The top 10 most abundant phyla and top 20 most abundant genera were selected for difference analysis. The phyla Proteobacteria, Actinobacteria, Fusobacteria, Bacillariophyta, Spirochaetes, Firmicutes, and Verrucomicrobia were found to have statistical differences in abundance among all groups (*P* < 0.05; **Figure 5A**). With the exception of *Prevotella* (characteristic sequence ASV-42), the remaining 15 dominant genera significantly differed among all groups (*P* < 0.05; **Figure 5B**). We also used a linear discriminate analysis effect size (LEfSe) to differentially analyze all taxonomic levels concurrently and robustly identify differential species among groups. Differential species identified were subjected to LDA analysis for estimating the effect size of differences between groups caused by abundance of these species. The taxonomic branch diagram in **Figure 5C** and the LDA bar chart in **Figure 5D** detail 21 statistically different species at all taxonomic levels among all groups in which14 species had LDA values of > 2. In oral microbiota, the marker species of samples obtained from normal weight children was *g_Alloprevotella* (LDA = 2.95; *P* < 0.001), while stable oral microbiota in obese group samples was characterized primarily by *g_Filifactor* (LDA = 3.98; *P* < 0.05) and *g_Butyrivibrio* (LDA = 2.54; *P* < 0.001). Among gut samples obtained from the oral cavity of normal weight children, the dominant marker species were characterized by *Bacteroidetes* (LDA = 5.11; *P* < 0.001), *f_Lachnospiraceae* (LDA = 5.10; *P* < 0.001), *g_Klebsiella* (LDA = 4.31; *P* < 0.05), *g_Citrobacter* (LDA = 3.94; *P* < 0.01), and *g_Eisenbergiella* (LDA = 3.85; *P* < 0.05). Among gut samples obtained from the oral cavity of children with obesity, dominant species included c_Clostridia (LDA = 5.40; *P* < 0.001), o_Clostridiales (LDA = 5.40; *P* < 0.001), f_Bacteroidaceae (LDA = 5.14; *P* < 0.001), f_Ruminococcaceae (LDA = 5.08; *P* < 0.001), *g_Faecalibacterium* (LDA = 5.02; *P* < 0.001), and *g_Tyzzerella* (LDA = 3.25; *P* < 0.01). The aforementioned data are detailed in **Figures 5C, D** and **Supplementary Table 4**. Differential species that passed the threshold were considered to be steady biomarkers among children with obesity and controls.

### Comparison of the metabolic characteristics

**Figure 6:**
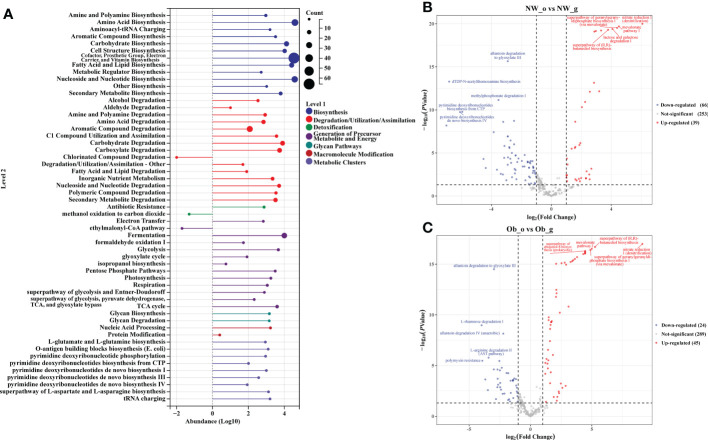
PICRUST2 analysis based on the MetaCyc Pathway Database. **(A)** Relative abundance of metabolic pathways at levels 1 and 2. **(B)** Differential analysis of metabolic pathways in oral and gut flora of controls. **(C)** Differential analysis of metabolic pathways in oral and gut flora of children with obesity.

**Figure 7:**
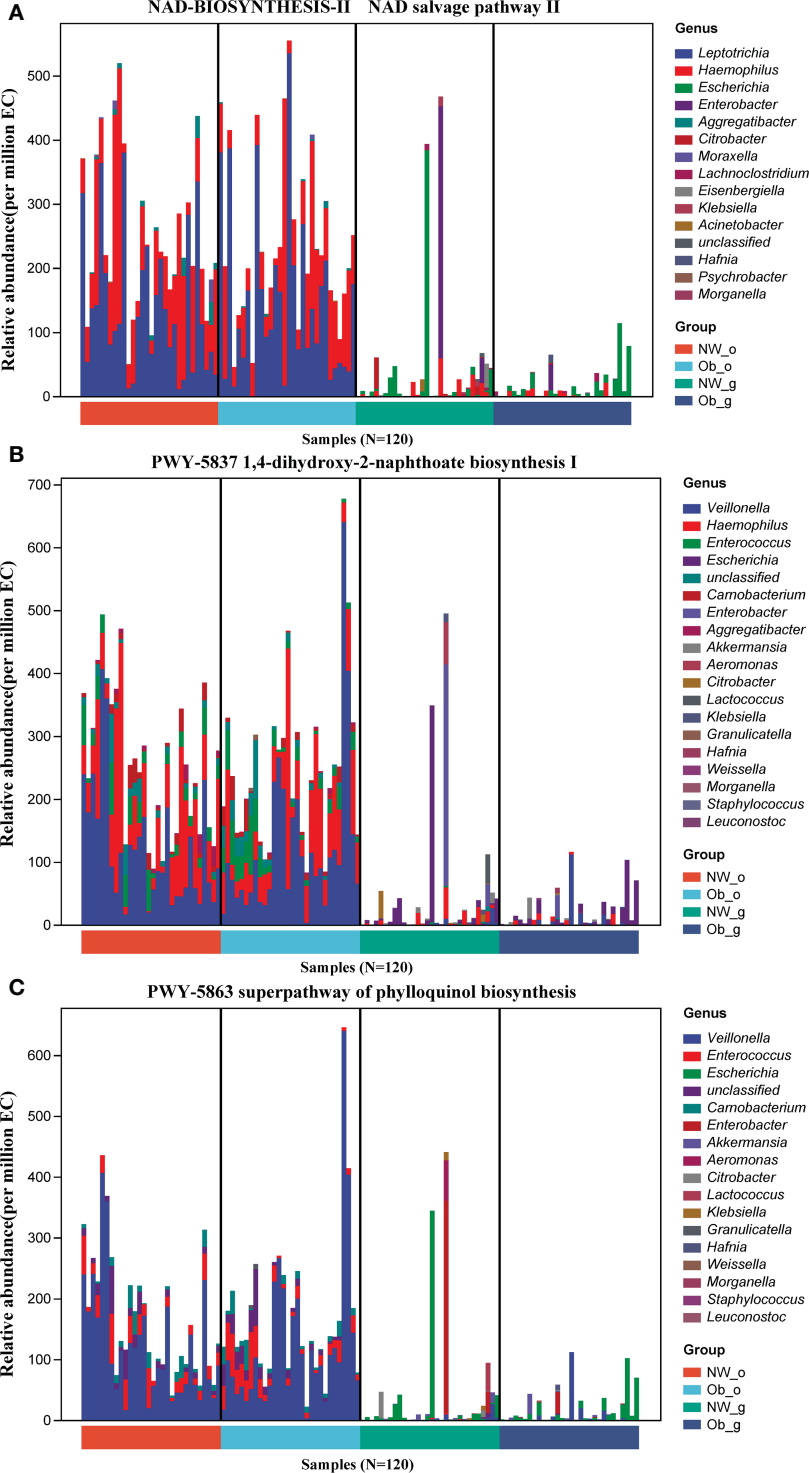
Composition of phylum involved in metabolic pathways. **(A)** NAD-BIOSYNTHESIS-II, NAD salvage pathway II. **(B)** PWY-5837, 1,4-dihydroxy-2-naphthoate biosynthesis I. **(C)** PWY-5863, superpathway of phylloquinol biosynthesis.

Prediction of the composition of flora genes or functional units was performed referencing the known microbial genome database MetaCyc. From the predicted results, the functional potential of oral and intestinal flora revealed more abundant metabolic pathways at primary levels, such as biosynthesis, degradation/utilization/assimilation, and generation of precursor metabolites and energy (**Figure 6A**). Secondary metabolic pathways, including amino acid biosynthesis, carbohydrate biosynthesis, cell structure biosynthesis (cofactor, prosthetic group, electron carrier, and vitamin biosynthesis), fatty acid and lipid biosynthesis, nucleoside and nucleotide biosynthesis, secondary metabolite biosynthesis, carbohydrate degradation, nucleoside and nucleotide degradation, fermentation, glycolysis, and the tricarboxylic acid (TCA) cycle showed richer relative abundance (**Figure 6A**). Stratified sample metabolic pathway abundance was used to analyze tertiary metabolic pathways. A total of 97 and 51 metabolic pathways significantly differed between oral and gut samples of children with obesity and controls, respectively (**Figures 6B, C**). Evaluation of the top 20 most differential metabolic pathways (screening criteria: absolute value of LogFC > 1, *P*-value < 0.05) revealed that when NW-o and Ob-o were considered as controls, seven bacterial metabolic pathways in NW-g samples and 16 in Ob-g samples were differentially up-regulated (**Tables 2**, **3**; *P* < 0.05). The only two pathways that were up-regulated only in normal weight children were PWY-6396 (superpathway of 2,3-butanediol biosynthesis) and REDCITCYC [TCA cycle VIII (helicobacter). Up-regulated pathways expressed only in children with obesity included NAD-BIOSYNTHESIS-II, PWY-5837, PWY-5863, P122-PWY, P124-PWY, PWY-5861, PWY-5897, PWY-5898, PWY-5899, PWY-5838, PWY-5840, and ARGORNPROST-PWY. We found that differences in metabolic functions of oral and intestinal flora in children with obesity reflected greater functional characteristics of the menaquinol superpathway. To understand which species encoded genes with certain functional potential, the composition of species involved in metabolic pathways was analyzed considering that all or most of these metabolic pathways could be independently analyzed according to species. Results revealed that the genera *Veillonella*, *Enterococcus*, *Haemophilus*, and *Leptotrichia* among oral flora in addition to *Escherichia* and *Enterobacter* among gut flora participated in the majority of differentially up-regulated metabolic pathways (**Figure 7**, *P* < 0.001); these organisms were most associated with the pathogenesis of obesity and relevant genus and species as detailed in **Supplementary Figure 7** and were deemed likely to encode genes relevant to these functions.

**Table 2:** Differential analysis of the top 20 metabolic pathways in oral and gut flora of normal weight children.

ID	Description	*P*-Value	FDR	LogFC	Type
PWY-5705	allantoin degradation to glyoxylate III	2.22E-16	7.99E-15	-2.911	Down
PWY-7315	dTDP-N-acetylthomosamine biosynthesis	5.24E-14	4.34E-11	-6.828	Down
PWY-3781	aerobic respiration I (cytochrome c)	7.15E-14	7.63E-13	2.853	Up
PWY-7391	isoprene biosynthesis II (engineered)	7.05E-13	1.40E-11	3.201	Up
PWY-7013	L-1,2-propanediol degradation	7.87E-13	2.07E-11	2.574	Up
PWY0-1533	methylphosphonate degradation I	7.14E-12	7.34E-11	-3.53	Down
PWY-6396	superpathway of 2,3-butanediol biosynthesis	1.21E-11	7.44E-11	2.257	Up
REDCITCYC	TCA cycle VIII (helicobacter)	4.88E-11	1.89E-10	2.041	Up
PWY-7210	pyrimidine deoxyribonucleotides biosynthesis from CTP	1.68E-10	9.25E-09	-5.953	Down
PWY-7198	pyrimidine deoxyribonucleotides *de novo* biosynthesis IV	1.78E-10	1.40E-08	-6.104	Down
PWY0-41	allantoin degradation IV (anaerobic)	1.88E-09	7.21E-08	-2.49	Down
P162-PWY	L-glutamate degradation V (via hydroxyglutarate)	2.38E-09	9.25E-09	1.598	Up
GLUCARDEG-PWY	D-glucarate degradation I	2.45E-09	1.25E-08	-3.216	Down
METH-ACETATE-PWY	methanogenesis from acetate	6.55E-09	2.72E-07	-7.01	Down
RHAMCAT-PWY	L-rhamnose degradation I	3.90E-08	5.60E-07	-3.853	Down
GALACTARDEG-PWY	D-galactarate degradation I	1.21E-07	5.81E-07	-2.911	Down
GLUCARGALACTSUPER-PWY	superpathway of D-glucarate and D-galactarate degradation	1.21E-07	5.81E-07	-2.911	Down
PWY-7242	D-fructuronate degradation	3.62E-07	3.14E-06	-2.725	Down
PWY-6383	mono-trans, poly-cis decaprenyl phosphate biosynthesis	7.15E-07	7.26E-06	1.941	Up
PWY-7446	Sulfoglycolysis	9.98E-07	8.11E-06	-2.486	Down

**Table 3:** Differential analysis of the top 20 metabolic pathways in oral and gut flora of children with obesity.

ID	Description	*P*-Value	FDR	LogFC	Type
PWY-3781	aerobic respiration I (cytochrome c)	1.11E-15	1.50E-14	2.902	Up
PWY-5705	allantoin degradation to glyoxylate III	3.11E-15	1.03E-13	-2.977	Down
NAD-BIOSYNTHESIS-II	NAD salvage pathway II	3.39E-13	2.01E-12	2.106	Up
PWY-5837	1,4-dihydroxy-2-naphthoate biosynthesis I	7.40E-13	1.46E-11	2.12	Up
PWY-5863	superpathway of phylloquinol biosynthesis	1.67E-12	3.10E-11	2.076	Up
PWY-7391	isoprene biosynthesis II (engineered)	1.54E-11	2.17E-09	3.099	Up
PWY-7013	L-1,2-propanediol degradation	6.14E-11	1.02E-09	2.387	Up
P122-PWY	heterolactic fermentation	1.76E-10	1.61E-09	1.483	Up
P124-PWY	Bifidobacterium shunt	3.30E-10	2.94E-09	1.475	Up
PWY-5861	superpathway of demethylmenaquinol-8 biosynthesis	4.16E-10	5.70E-09	1.735	Up
PWY-5897	superpathway of menaquinol-11 biosynthesis	5.17E-10	6.37E-09	1.682	Up
PWY-5898	superpathway of menaquinol-12 biosynthesis	5.17E-10	6.37E-09	1.682	Up
PWY-5899	superpathway of menaquinol-13 biosynthesis	5.17E-10	6.37E-09	1.682	Up
PWY-5838	superpathway of menaquinol-8 biosynthesis I	9.91E-10	1.17E-08	1.632	Up
RHAMCAT-PWY	L-rhamnose degradation I	1.07E-09	5.70E-09	-3.986	Down
PWY-5840	superpathway of menaquinol-7 biosynthesis	2.42E-09	2.72E-08	1.596	Up
PWY0-41	allantoin degradation IV (anaerobic)	7.05E-09	3.08E-07	-2.219	Down
KDO-NAGLIPASYN-PWY	superpathway of (Kdo)2-lipid A biosynthesis	3.88E-08	2.53E-07	1.582	Up
ARGORNPROST-PWY	arginine, ornithine and proline interconversion	2.85E-07	2.16E-06	1.427	Up
PWY-7373	superpathway of demethylmenaquinol-6 biosynthesis II	9.06E-07	2.76E-05	1.823	Up

## Discussions

Prior studies have confirmed that changes in the oral or gut flora can reflect the characteristics of specific health conditions (Xiao et al., 2020). Many studies have demonstrated that the oral and gut microbiota are associated with obesity. Researchers have found that that the diversity of saliva and subgingival plaque bacteria in children decreases as body mass index (BMI) increases (Janem et al., 2017). A study (Wu et al., 2018) comparing saliva among obese and normal-weight adults reported that the microbial diversity and richness of saliva obtained from obese individuals with good periodontal health was significantly lower than that of controls. The abundance of *Prevotella*, *Granulicatella*, gastric *Streptococcus*, *Solobacterium*, *Catonella*, and *Mogibacterium* in samples obtained from obese patients was higher than that of the control group, whereas members of the genus *Staphylococcus* were less abundant in the obese group. The oral microbiome also modulates the composition of the gut microbiome. This suggests that the composition of the oral microbiome can affect the composition of the gut microbiome *via* immunomodulation and thereby potentially exacerbate the severity of obesity. Both microbiomes form a complex oral-gut cross-talk system that participates in the regulation of host health and disease (Finlay et al., 2019; Guo et al., 2019). Therefore, studies of the oral and gut microbiota in the context of obesity are needed to explain the relationship between the obesity epidemic and the microbiota.

In this study, we explored the characteristics of the oral and intestinal microbiota in children with obesity aged 3-5 years to determine any potential impact of the oral or intestinal microbiome on obesity, as investigated *via* comparative analysis. First, we determined alpha diversity and beta diversity indices to distinguish the differences in the microbial communities of obese children and normal-weight controls. In this study, the alpha diversity indices, including Chao1, observed species, and Shannon, revealed statistically significant differences in the oral and gut microbial composition of children in different BMI categories. Although obese children had a more diverse oral microbiota, the diversity of their intestinal microbiome was lower (Chao1: *P*<0.001; observed species: *P*<0.001; Shannon: *P*<0.001), consistent with research indicating that the composition of the gut microbiota changes and that the microbial diversity decreases in obese humans and rats (Petriz et al., 2014). Beta diversity, which reflects the heterogeneity of the microbiota between samples, was examined using weighted-UniFrac values to calculate and compare differences in the microbiota composition of each group. These analyses revealed an apparent clustering pattern in the oral and gut microbiota, but there was no obvious difference between the obese and normal-weight groups. The observed differences in diversity indices suggest that the structure of the microbiota changes significantly with changes in body weight and that these changes may be associated with the occurrence of obesity.

The dominant bacterial phyla and genera in oral and intestinal samples reported in other studies include Firmicutes, Proteobacteria, and Bacteroidetes, *Neisseria*, and *Streptococcus*, similar to our study. At the phylum level, a low microbial diversity and high Firmicutes/Bacteroidetes (F/B) ratio of gut microbiota are often cited as distinguishing features of adult and adolescent obesity. Researchers have found that compared to persons of normal weight, individuals with obesity have a higher F/B ratio among the oral and gut bacteria (Mathur and Barlow, 2015; Castaner et al., 2018; Castaner et al., 2018; Sohail et al., 2019). Our results show that Firmicutes derived from either the oral cavity or fecal samples were more abundant in samples obtained from children with obesity. However, bacteria of the Bacteroidetes phylum were less abundant in samples obtained from normal-weight children. Thus, in children with obesity, the F/B ratio was higher compared with that among children of normal weight, in terms of both oral and fecal samples. Craig et al. (2018) reported that rapid weight gain is positively correlated with F/B ratio, an indicator of obesity, in the oral microbiota of children. However, other relevant studies reported no significant difference in F/B ratio between the normal-weight and obesity groups (Schwiertz et al., 2010; Karlsson et al., 2012). Such differences can be attributed to differences between subjects, lifestyle, eating habits, and complexity of the microbiota.

Due to social isolation and changes in lifestyle, children have been one of the groups most heavily impacted by the COVID-19 pandemic over the past 3 years. Social isolation increase the potential for detrimental behaviors, such as consumption of a sugar-rich diet and engaging in a sedentary lifestyle, which may have led to an increase in the risk of early childhood caries and obesity during the COVID-19 pandemic. Interestingly, *Streptococcus mutans* and *Lactobacillus* spp., the two primary caries-associated pathogens belonging to the phylum Firmicutes, are more abundant in the gut microbiota of children with obesity, suggesting that oral Firmicutes may reflect the gut condition of preschool-aged children with obesity (Reis et al., 2022). However, other studies have suggested that the relationship between dental caries in primary teeth and obesity can vary based on the definitions of obesity and dental caries (Piovesan et al., 2022).

The oral microbiota of young children was extensively studied by Coker et al. (2022). In that study, analysis of the community composition of the salivary microbiota by growth status indicated that the majority of the saliva microbiota consisted of members of the Firmicutes, followed by Proteobacteria, Actinobacteria, and Bacteroidetes. The top genera in terms of relative abundance were *Streptococcus* and *Neisseria*, a finding that was consistent with our research. Furthermore, certain taxa, such as *Actinomyces odontolyticus* and *Prevotella melaninogenica*, were associated with decreased weight or growth, and *Streptococcus mitis* and *Corynebacterium matruchotii* were associated with increased growth across anthropometric parameters. Coker et al. (Coker et al., 2022) also reported that both rapid weight gain between 0 and 2 years of age and weight-to-length ratio were associated with BMI at 3-4 years of age among both males and females, a finding that was in accordance with those of a study of 2-year-old children by Craig et al. (Craig et al., 2018), who reported that children who gain weight rapidly before the age of 2 years are more likely to be obese later in childhood and adulthood. The oral microbiota is thought to mediate obesity signals even earlier than the gut microbiota. A study of the gut microbiota in the first 2 years of life indicated an association between the infant gut microbiota and later BMI. The study’s authors offered preliminary evidence that the infant gut microbiota, particularly at 2 years of age, could help identify children at risk for obesity (Stanislawski et al., 2018). If the above findings could be confirmed in a larger group of preschool children, the ability to clinically identify children at risk for obesity would be greatly facilitated.

Over-representation of Prevotellaceae has been proposed as a marker of microbial dysbiosis predisposing to inflammation and metabolic disease. Studies by Könönen et al. (Könönen and Gursoy, 2022; Könönen et al., 2022) reported that in the oropharynx of young adults, tonsillar crypts are colonized by a variety of *Prevotella* species, namely, *P. pallens* and *P. salivae*, which are typical oral species. *Prevotella pallens* was reported as a ubiquitous species in the oral cavity, where it colonizes mucosal surfaces of infants from the early months of life onwards. Similar clones of *P. pallens* recovered from maternal saliva and oral samples of their young children were mainly derived from periodontally healthy mothers. In addition, *Prevotella copri* is known to inhabit the gut. All of these findings were similar to those of our study. **Figure 4A** shows that *P. salivae* and *P. pallens* seemed to be more abundant in the salivary microbiome of normal-weight controls, whereas *P. copri* was more abundant in the gut microbiome of normal-weight controls. In our study, *P. oulorum* was enriched in saliva samples of normal-weight controls (**Figure 5C**). We also identified an oral cavity biomarker among normal-weight controls, namely *Alloprevotella* (**Figure 5D**), which is a short-chain fatty acid–producing and anti-inflammatory bacterium that is almost absent in hypertensive patients. Studies also have reported increases in the number of some *Prevotella* species in the setting of localized and systemic diseases, such as periodontitis, bacterial vaginosis, rheumatoid arthritis, and metabolic diseases (Larsen, 2017). Another study proposed the Prevotellaceae family as a marker of microbial symbiosis (Furet et al., 2010); however, in our study, no *Prevotella* were found to play a role as potentially harmful agents in the oral/gut microbiota of children with obesity.

We also identified several steady biomarkers among obese children using LEfSe analysis. Genus-level LEfSe analysis revealed higher proportions of *Filifactor* and *Butyrivibrio* in the oral microbiota of obese children, whereas the fecal microbiota of obese children was more enriched in *Faecalibacterium*, *Tyzzerella*, and *Klebsiella*. To investigate the relationship between oral/gut microbiome functions and childhood obesity, we used PICRUSt to predict the potential metagenomes. We found that with the change in relative abundance of microbiota in children with obesity, the functions of these organisms also changed correspondingly. Compared with normal-weight controls, the differences in the metabolic functions of the oral and intestinal flora in obese children were reflected more clearly in the functional characteristics of the NAD salvage pathway, naphthoate, phylloquinol synthesis, and other biosynthesis and associated super-pathways. The genera *Veillonella* and *Enterococcus* were found to be involved in differentially up-regulated (*P*<0.001) metabolic pathways. Although these findings regarding biomarkers and functions could aid in predicting obesity in childhood, a larger sample size would facilitate more in-depth research in this regard.

The mechanism by which the oral flora influences human health and obesity *via* the intestinal microbiome remains unclear. *Ruminococcus* is a fermentative bacteria that degrades food fibers that cannot be digested by the human body into absorbable short-chain fatty acids which increase energy intake through intestinal absorption (Turnbaugh et al., 2006). Increased *Klebsiella* abundance in the oral cavity can lead to disruption of the oral microbiome, which in turn can increase the permeability of the intestinal mucosa and aggravate the pathogenesis of enteritis *via* inflammasome activation (Kitamoto et al., 2020). This, in turn, can lead to increased levels of circulating lipopolysaccharide and contribute to the development of obesity (Cani et al., 2008). Some studies have suggested that intestinal microbes such as *Lactobacillus* and *Bifidobacterium* dropped act to trigger triglyceride accumulation in host adipocytes through various regulatory mechanisms (Sun et al., 2018). The intestinal microbiota acts to reduce hepatic fatty acid oxidation *via* inhibition of adenosine monophosphate kinase, and inhibition of this enzyme in the liver and muscle tissue likely causes an increase in accumulation of body fat (Zama et al., 2020). However, few reports are available regarding the relationship between obesity and *Filifactor*, *Butyrivibrio*, *Faecalibacterium*, and *Tyzzerella*, highlighting the need for further research. Our study provides evidence confirming that increases and decreases in the abundance of key biomarker species are closely associated with the development of obesity.

In this study, we analyzed the characteristics of the oral and intestinal microbiome in children aged 3-5 years to provide a preliminary assessment of the relationship between the microbiota and obesity. Our findings indicate that microbial diversity as well as the structure of the oral and gut microbiomes differ significantly between healthy children and children with obesity and provide new evidence that changes in the microbiome contribute to the risk of obesity. Importantly, we identified several steady biomarkers and microbiome functions among obese children that may aid in the development of methods to identify children susceptible to obesity.

However, our study has several limitations. First, this was a cross-sectional study and could not provide evidence of a causal effect between changes in the oral/gut microbiota and obesity. As an association exists between the gut microbiota and child growth, it is possible that tracking a specific study population over time and exploring the interactions between the oral and gut microbiomes in young children could reveal important changes in the bacterial community structure and provide valuable information that will help us better understand the course of obesity.

## Conclusion

The current study showed that the diversity and structure of the oral and gut microbiota in children with obesity differ significantly compared with normal-weight children. Oral and intestinal dysbiosis are closely related to obesity. We also identified various bacterial metabolic pathways that could be involved in obesity development and found that the expansion of some bacteria in saliva and feces could play a role in the development of obesity *via* immune-inflammatory processes. However, at present, there are still many inconsistencies in the data, and studies with larger numbers of samples will be needed to confirm the causality between specific intestinal bacterial species and obesity. In addition, the relevant mechanisms need to be explored in greater detail. Microflora transplantation has emerged as a hot topic in obesity research. More studies examining the characteristics of the oral and intestinal microflora of obese children would be very helpful in the development of approaches to predict and treat childhood obesity.

## Data availability statement

The data presented in the study are deposited in the National Center for Biotechnology Information (NCBI) repository, accession number PRJNA903817.

## Ethics statement

The studies involving human participants were reviewed and approved by Research and Ethics Committee of the First Affiliated Hospital of Xinjiang Medical University. The patients/participants provided their written informed consent to participate in this study.

## Author contributions

Conceived and designed the study: JZ, TM, and ZW. Participated in investigation: TM, JL and CS. Performed formal analysis: TM, ZW. Collected the resources: TM, JL, ZW, and CS. Curated the data: TM, JL and CS. Wrote the manuscript: TM and ZW. Supervised the study: JZ, TM, and AA. All authors contributed to the article and approved the submitted version.
