# Supplementary material for: Characterization of the oral and gut microbiome in children with obesity aged 3 to 5 years
Source: Front Cell Infect Microbiol. 2023 Mar 29;13:1102650. doi: 10.3389/fcimb.2023.1102650 (PMC10090557; doi:10.3389/fcimb.2023.1102650)
Supplement: Supplementary file 1 [file DataSheet_1.pdf]

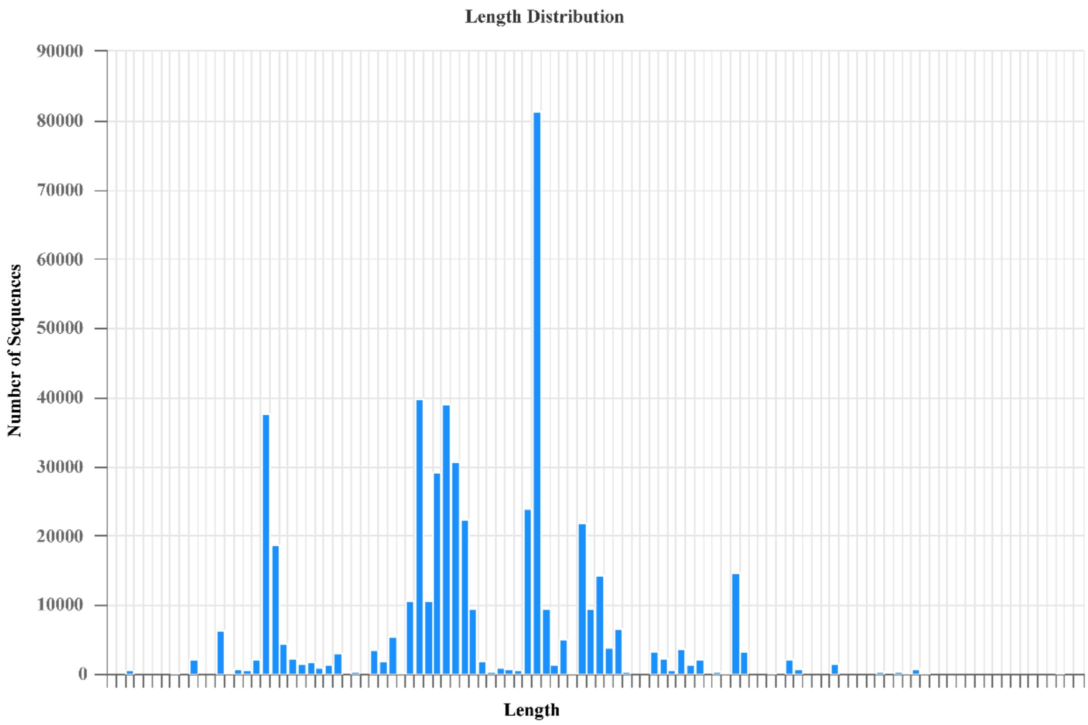

Supplementary Figure 1 The distribution of valid sequences length.

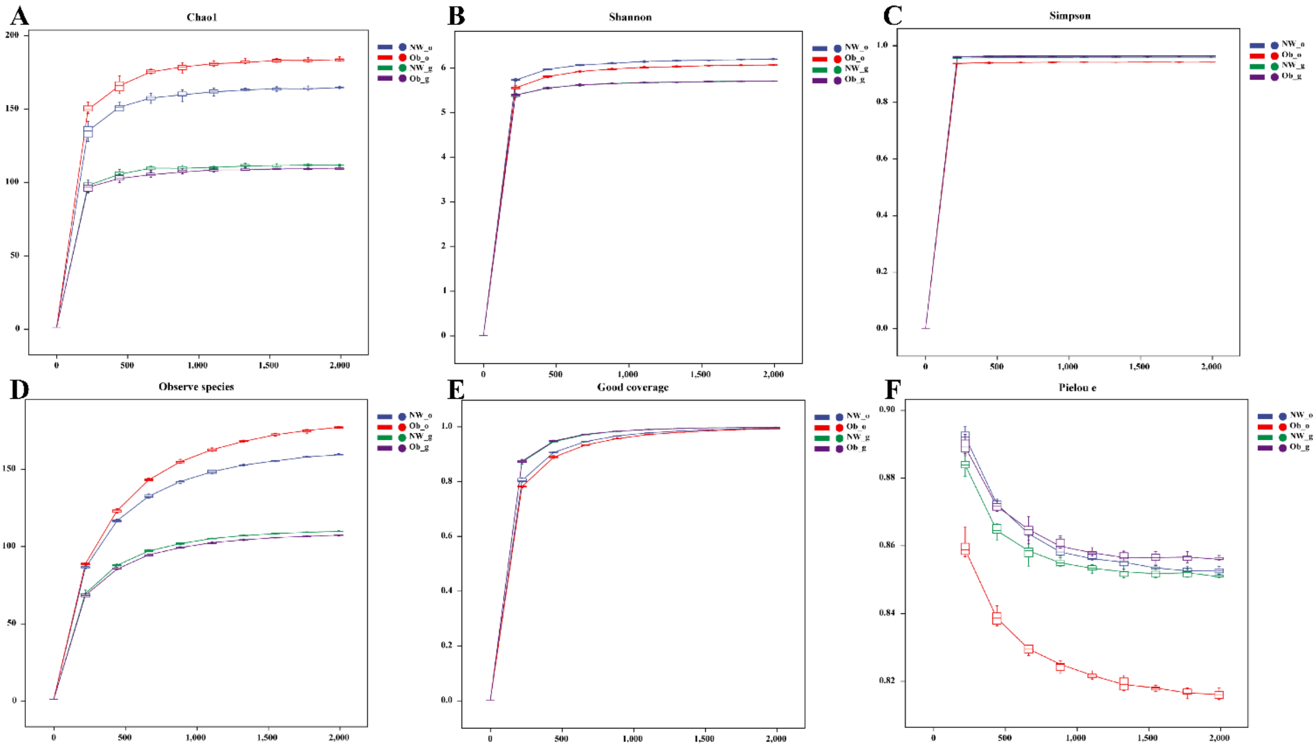

Supplementary Figure 2 Rarefaction curve of alpha diversity index. Alpha diversity index including (A) Chao 1; (B) Shannoon; (C) Simpson; (D) Observed specie; (E) Good coverage; (F) Pielou_e.

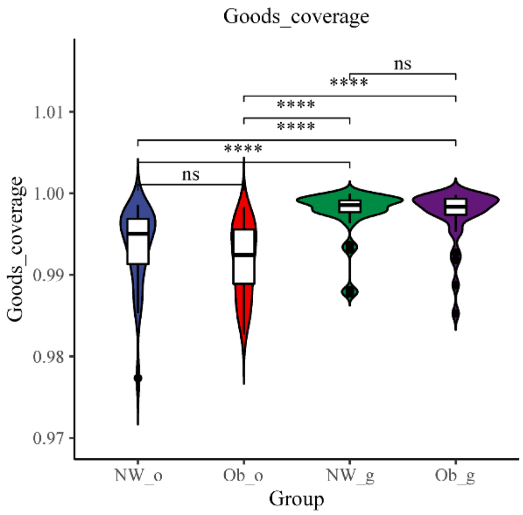

Supplementary Figure 3 The violin Diagram of Alpha diversity analysis. Alpha diversity index Goods coverage.

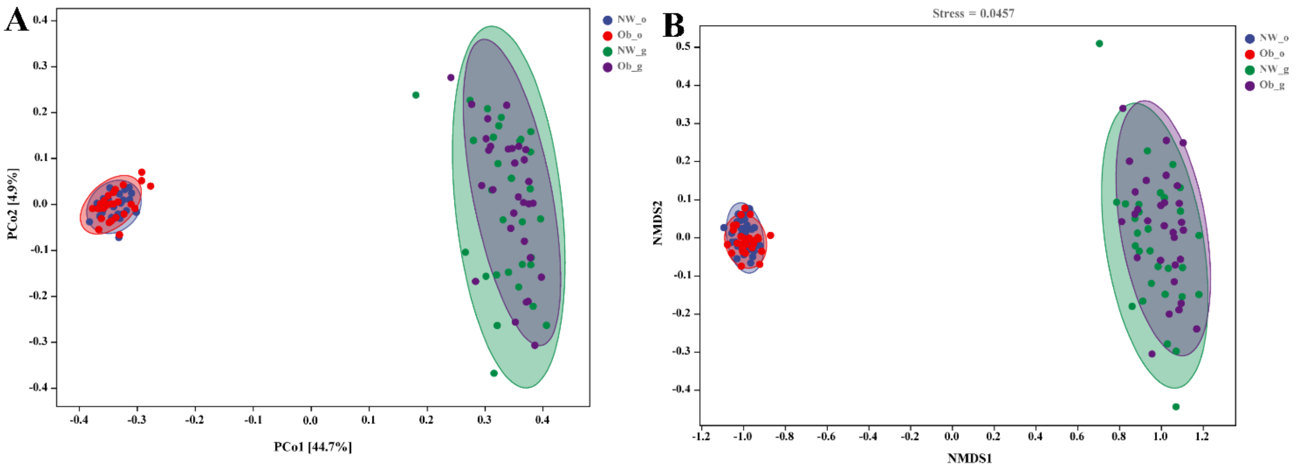

Supplementary Figure 4 Beta diversity analysis based on unweighted-unifrac distance. (A) Principal coordinate analysis (PCoA) of bacterial communities from the four groups. (B) Nonmetric multidimensional scaling (NMDS) analysis of bacterial β diversity from the four groups.

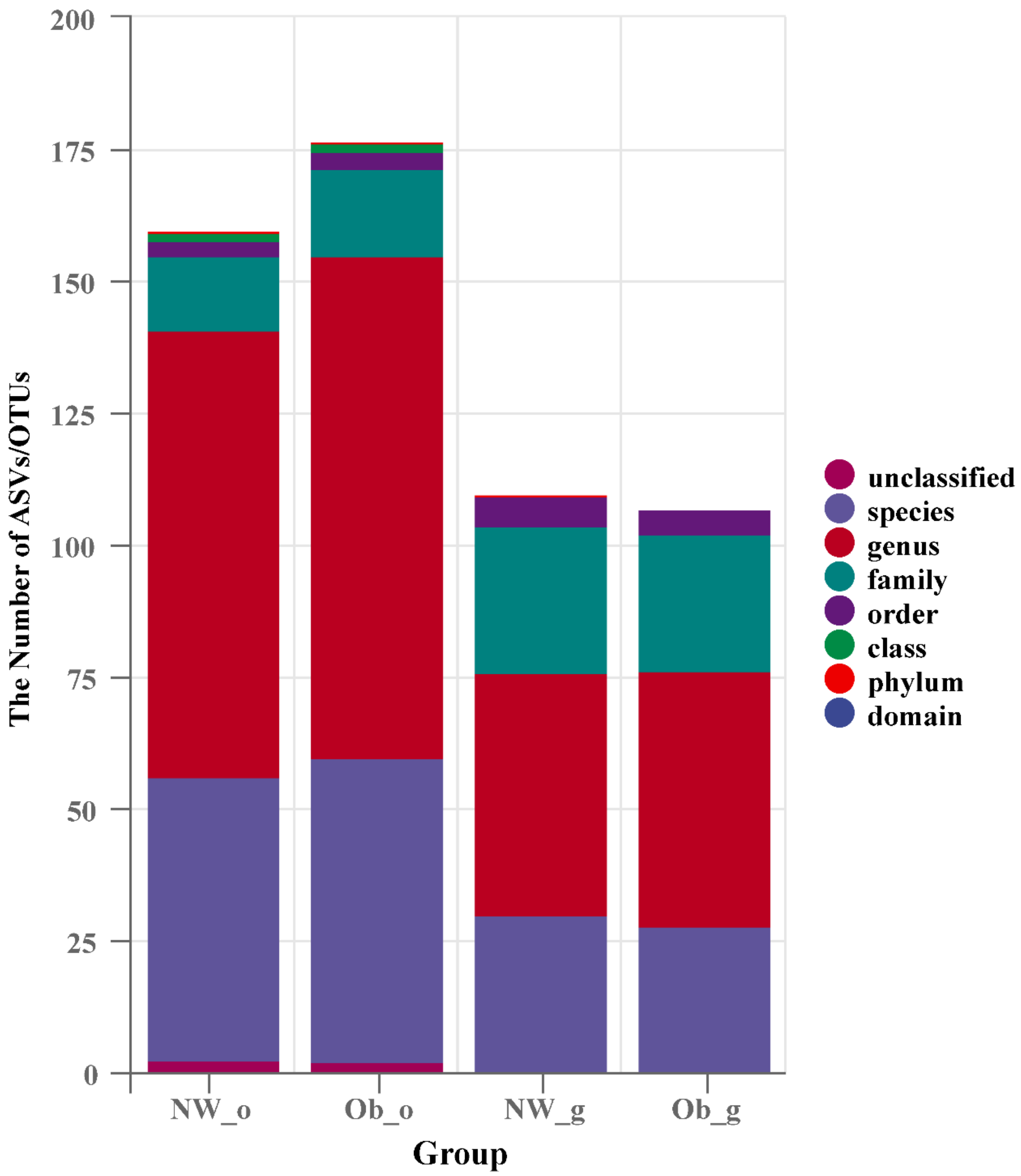

Supplementary Figure 5 Statistical chart of the results of classification of ASVs.

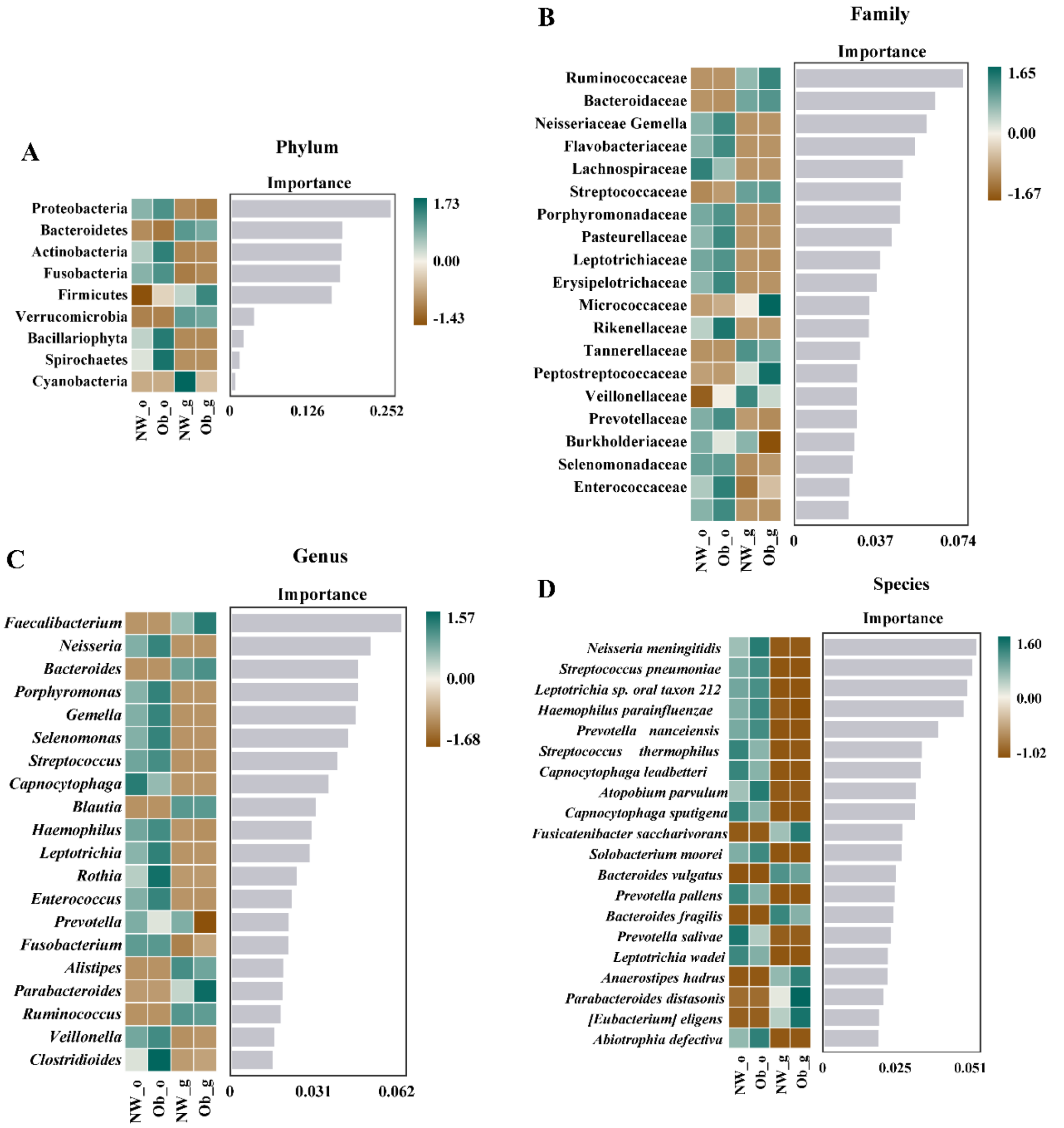

Supplementary Figure 6 random forests at phylum, family, genus and species level. The vertical axis is the taxon name at the phylum, family, genus, and species level; The heat map shows the abundance of species in each group; The bar graph is the importance score of the species to the classifier model, the importance of species to the model decreases from top to bottom.

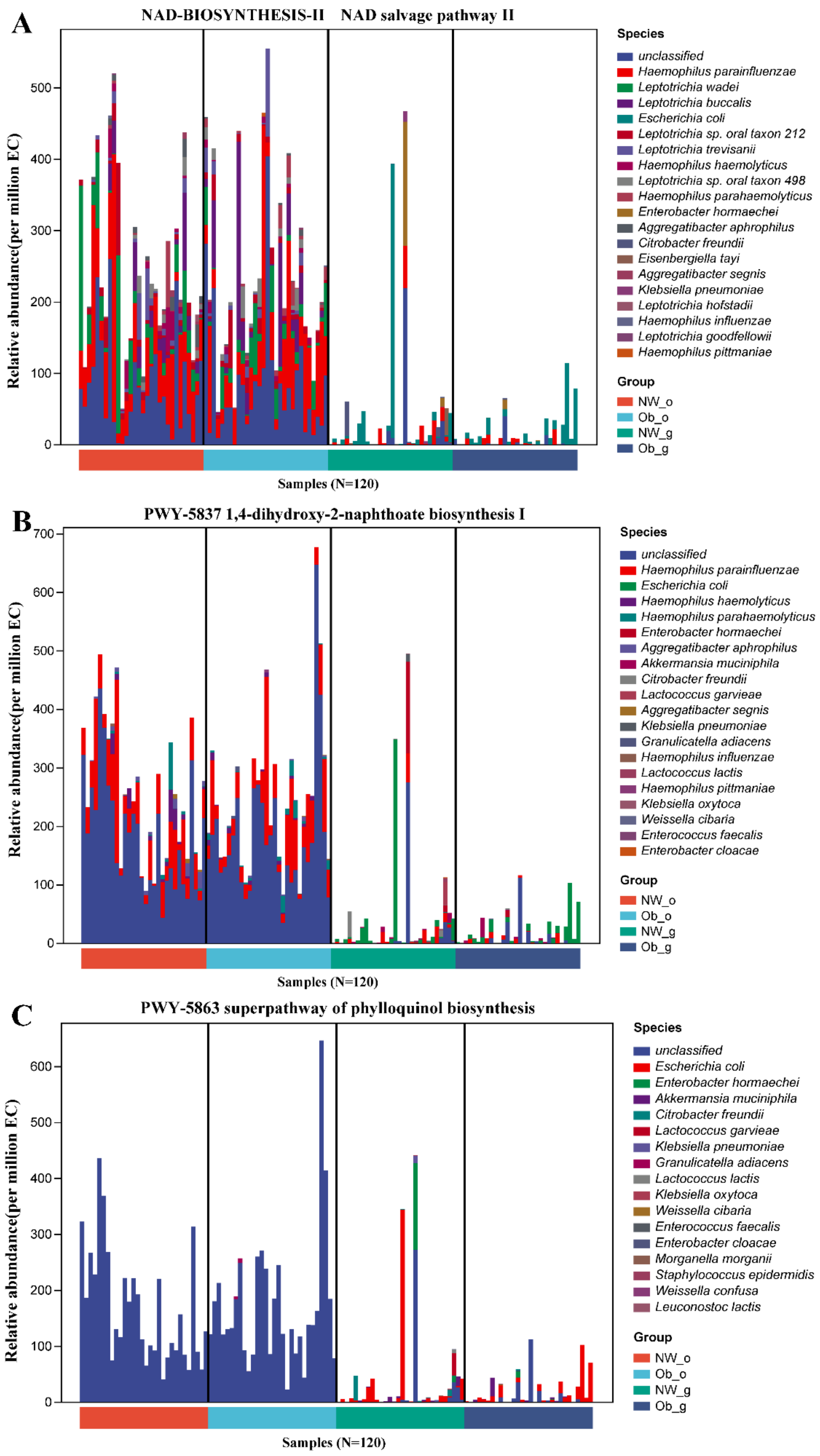

Supplementary Figure 7 Composition of species involved in metabolic pathways.
